# What Is the Relationship Between Empathy and Mental Health in Preschool Teachers: The Role of Teaching Experience

**DOI:** 10.3389/fpsyg.2020.01366

**Published:** 2020-07-07

**Authors:** Heqing Huang, Yanchun Liu, Yanjie Su

**Affiliations:** ^1^College of Preschool Education, Capital Normal University, Beijing, China; ^2^College of Education Science, Hubei Normal University, Huangshi, China; ^3^School of Psychological and Cognitive Sciences, Peking University, Beijing, China

**Keywords:** mental health, empathy, teaching experience, preschool teachers, empathic concern, personal distress, perspective taking

## Abstract

The present study aimed to delineate the characteristics of empathy and mental health in preschool teachers and to examine the role of empathy in preschool teachers’ mental health. The sample in this study consisted of 4348 preschool teachers, who were divided into four groups according to their years of teaching experience (less than 2, 2–5, 5–10, and more than 10 years). The Chinese version of the Symptom Checklist 90 was used to measure the mental health symptoms of the participants, and the Chinese version of the interpersonal reactivity index was employed to assess various aspects of the participants’ empathy. The results indicated that most symptoms increased as teaching experience increased, independent of the effect of age. The study also found that the four dimensions of empathy showed different trends across the four teaching experience groups: fantasy remained stable, empathic concerns and perspective taking showed decreasing trends, and personal distress showed an increasing trend. Moreover, the present research found a relatively complex relationship between empathy and mental health in preschool teachers: whereas fantasy and personal distress positively predicted mental health symptoms in preschool teachers, perspective taking and empathic concern negatively correlated with most of the symptoms. It seems that empathy contains both risk and protective factors for individuals’ mental health, and these factors are affected by years of teaching experience.

## Introduction

The preschool teachers’ job is an emotional effort (Lee et al., 2016). Throughout their whole career life span, preschool teachers were surrounded by various emotions, and the teachers have to feel, understand, and react to others’ emotions appropriately (Ahmetoglu and Acar, 2016; Gaines et al., 2019). Empathy, the ability to connect others’ feeling without losing himself/herself (Chiu and Yeh, 2017), is found to play an important role in preschool education (Ahmetoglu and Acar, 2016). Research has suggested that empathy is one of the crucial factors that influence how successful preschool teachers are in leading their personal and professional lives (Vucinic et al., 2020). Therefore, empathy is regarded as one of the core contents of teachers’ professional development (Bullough, 2019), and significant differences were found between new and experienced teachers (Mevarech and Maskit, 2015).

As the emotional nature of teaching, empathy might be associated with teachers’ mental health symptoms (Mérida-López et al., 2017). It is also possible that during the career development of preschool teachers, empathy differently correlated with mental health across different development stages. However, research on how empathy affects preschool teachers’ mental health is sparse. The present study aimed to examine the relationship between empathy and mental health, and the role of teaching experience in the relationship.

### Preschool Teachers’ Mental Health Status

According to the World Health Organization (2006), mental health is a state of well-being that enables individuals to successfully cope with the normal stresses of life and work productively and contribute to their community. Mental health and its assessment are one of the broadest and most complicated issues in psychology given the many contributing factors (Keyes et al., 2002; Franken et al., 2018), and adverse symptoms, for example, depression, anxiety, and somatization, reflect aspects of mental health status (Chekroud et al., 2017).

Teaching has one of the highest rates of stress and burnout, which interfere with mental health (Grayson and Alvarez, 2008; Miller et al., 2019). The issue of mental health and well-being in teachers is critical not only for their teaching performance but also for the students (Dods, 2016; Miller et al., 2019). On the one hand, plenty of research has reported a positive relationship between teachers’ mental health status and their job satisfaction and efficacy beliefs (Allan et al., 2016) and negative relationships between mental health status and burnout and depression (Jennings et al., 2017; Capone and Petrillo, 2018), which sometimes even leading to teachers’ leaving their professions (Bowles and Arnup, 2016). On the other hand, teachers’ mental health is correlated with the students’ developmental outcomes (McLean and Connor, 2018). A recent study found that secondary teachers’ better mental health was associated with students’ better well-being and lower psychological distress (Harding et al., 2019).

In regard to the status of preschool teachers’ mental health, the results are mixed. Although a recent meta-analysis suggested that in China, preschool teachers’ mental health conditions are at least as good as, if not better than, those of other kinds of teachers, for example, primary and secondary teachers (Fan et al., 2016); other research has reported different results, indicating that education is a work context in which professionals (teachers) are likely to suffer from burnout, which may be associated with low levels of mental health (Zhang et al., 2014). Compared with K-12 teachers, preschool teachers scored higher than Chinese national controls on the SCL-90 (Chen, 2009), but more detailed information concerning preschool teachers is absent. Accordingly, the present study aims to determine the status of preschool teachers’ mental health.

### Preschool Teachers’ Empathy and Its Role in Their Mental Health

Empathy, the capacity to feel and understand the emotion of others, is widely accepted as a multifaceted construct that contains both cognitive and affective components (Preston and de Waal, 2002; Buck et al., 2017). Among a variety of empathy multicomponent assumptions, Davis’s (1983) point of view is representative. According to him, empathy contains four components: personal distress, empathic concern, fantasy, and perspective taking. Among these components, perspective taking, which is the trend and ability to walk in others’ shoes, is a typical cognitive component. Although both personal distress and empathic concern are regarded as emotional components, they are believed to have opposite functions, with personal distress making individuals focus on themselves and empathic concern enabling individuals to attend to the others. In addition, fantasy is individuals’ abilities and trends to transpose themselves into fictional situations. Another representative multicomponent assumption is Preston and de Waal’s (2002) *russian doll model of empathy*, which assumed that empathy is a layered structure, with personal distress and emotional contagion in its emotional core, the more advanced form of empathy (a mixture of emotional and cognitive components, e.g., empathic concern) in the middle layer, and cognitive component (e.g., perspective taking) in the outer layer. Decety and Lamm (2006) also built a social neuroscience model of empathy, which argues that empathy involves both emotion sharing (bottom-up information processing) and the regulation and modulation to this experience (top-down information processing); and the feeling of empathic concern is the outcome of the experience being regulated.

Empathy enables teachers to feel and understand what the child is feeling, communicate about the feeling with him/her, and then responding in ways that meet his/her needs. In preschool settings, each aspect of empathy plays an important role in guiding interactions between the teacher and child. For example, when encountering a crying child, a preschool teacher may have multiple empathy reactions: she/he may feel anxious and upset and may urge the child to stop crying (the feeling of personal distress), she/he may feel warm and soft-hearted and want to help the child (the feeling of empathic concern), or she/he may also consider cognitively what has happened to the child (perspective taking). In addition, the preschool teacher’s responding with the child may also be automatically affected by the fictional characters in books or films that she/he have encountered.

As early education settings are highly emotionally demanding, empathy is usually regarded as a trait and a skill necessary for the teaching and caring for children, and teacher empathy emerges as a highly desirable trait (Peck et al., 2015). However, what is the role of empathy in teachers’ well-beings and mental health? Previous research concerning the relationship between empathy and mental health is mixed. Both positive and negative relationships were found between empathy and mental health-related issues.

On the one hand, empathy was proved to correlated with individuals’ better social relationship (Coutinho et al., 2014), higher well-being (Carnicer and Calderón, 2014), greater professional satisfaction (Halpern, 2003), and emotional self-efficacy (Goroshit and Hen, 2014), and all these issues have a close relationship with one’s mental health (Wacker and Dziobek, 2018). There are also evidence that empathy is a protective factor of burnout in physicians (Lamothe et al., 2014; Thirioux et al., 2016). Empathy can also improve one’s emotional state, as empathy is proposed to be a method of interpersonal emotion regulation, which have proved to be healthy and efficient (Zaki, 2019). On the other hand, researchers have also noticed the relationship between empathy and mental health symptoms. For example, empathy was found to be positively related with depression (Zhang et al., 2013) and anxiety (Fulton, 2012; Gambin and Sharp, 2018). The risk role of empathy in mental health was especially obvious in helping professionals; for example, empathy was found to have a close relationship with burnout and secondary trauma in caregivers (Kim, 2017), physicians (Tone and Tully, 2014), social workers (Macritchie and Leibowitz, 2010), and so on.

The mixed results may stem from the complication of both empathy and mental health. Although the multicomponent assumption of empathy has been wildly accepted, most of the research mentioned above does not especially examine how different dimensions of empathy correlated with mental health-related issues. Further investigation of the studies mentioned above implied that most of the research supporting the protective effect of empathy in mental health is biased to the cognitive aspect of empathy (Dekel et al., 2017), whereas research found that a risk role of empathy in mental health problems is focused on the emotional aspect of empathy (Williams, 1989; Dekel et al., 2017). Accordingly, we hypothesize that emotional empathy is prone to mental health problems whereas cognitive empathy plays a protective role in the among this research.

In this research, we will examine this complex relationship between empathy and mental health. Moreover, we also want to examine this relationship in preschool teaching profession, which is a typical helping profession (Erera, 1997) and a kind of emotional labor (Philipp and Schüpbach, 2010).

### The Role of Teaching Experience

Teaching experience is a critical variable that should not be neglected when considering the mental health and empathy of teachers. Researchers have found that teaching experience, not age, correlates with variables related to empathy and mental health in educational settings. Researchers have found a relationship between working experience and mental health in both educational and non-educational contexts. For example, in physicians, professional experience negatively related with mental health (Yekta et al., 2014; Dai et al., 2015); in teachers, teaching experience contributed to the negative emotions concerning their professional identities (Mevarech and Maskit, 2015). Experience is also found to be related with empathy. For example, in psychiatrists, subjects with more years of experience had lower empathic concern scores (Santamaría-García et al., 2017); in preservice preschool teachers, years of study is also found to correlate with various dimensions of empathy (Huang et al., 2018).

Katz (1972) has emphasized that teaching experience is one of the core variables of preschool teachers’ characters. Based on Katz’s theoretical frame, the growth of preschool teachers generally proceeds in four stages: survival, consolidation, renewal, and maturity. It is theoretically assumed that in different developmental stages, teachers have different psychological characteristics; however, from the empirical perspective, to date, we do not know how teaching experience affects preschool teachers’ mental health and empathy across Katz’s four stages. We hypothesize that in different stages of teacher development, empathy plays different roles in preschool teachers’ mental health.

## The Present Study

Briefly, the purpose of the present study is to investigate the dynamic aspects of empathy and mental health and their relationship, as well as the effect of teaching experience on these factors. There are two hypotheses in this study. The first hypothesis is that empathy dimensions correlate with mental health differently, emotional dimensions may be risk factors, and cognitive dimensions may be protective factors to the teachers’ mental health. The second hypothesis is that teaching experience plays critical roles in the relationship between mental health and empathy of preschool teachers.

## Materials and Methods

### Participants

The original sample consisted of 4343 preschool teachers, as 42 respondents’ data were deleted from the analyses because of incomplete information, and the final sample consisted of 4301 preschool teachers from 544 preschools. These preschools were distributed throughout the 19 administrative districts in Beijing City and its surroundings. All the preschools were public, and in 2015, when the data for the present study were collected, there were 913 public preschools and approximately 21,000 preschool teachers in the Beijing area (Beijing Municipal Government, 2017); thus, approximately 51.58% of the preschools and 20.48% of preschool teachers participated in the present study. The participating preschools were randomly selected. According to the Chinese Ministry of Education (2012), all public preschools in China can be classified into four categories—model preschools, first-class preschools, second-class preschools, and third-class preschools—according to more than 10 criteria, for example, the preschools’ site size and hardware level, numbers of students and teaching staff, and the quality of courses and activities. The local educational authorities conduct comprehensive assessments of each preschool according to these criteria every 3 years. In the present study, 11% of the preschool teachers were from the model preschools, 22% were from the first-class preschools, and 31% were from the second-class preschools. The teachers in each preschool were selected randomly and asked to complete the questionnaires through mobile phones or computers.

The age range of the final sample was 18.10 to 59.06 years, with a mean age of 30.07 years (SD = 8.44), and the participants’ teaching experience ranged from 0.12 to 44.00 years (*M* = 8.11; SD = 8.10). To compare the teaching experience of the preschool teachers, we divided the sample into four groups according to Katz’s (1972) theory concerning preschool teachers’ career development: less than 2 years of teaching experience (*n* = 1237), 2–5 years of teaching experience (*n* = 1138), 5–10 years of teaching experience (*n* = 638), and more than 10 years of teaching experience (*n* = 1294). The detailed demographic characteristics of the respondents are illustrated in Table 1. In both the whole sample and each teaching experience group, most of the participants were female (93–98.4%) and ethnic minorities (94.1–94.9). The other demographic information of the participants is presented in Table 1.

**TABLE 1:** Demographic variables of the participants.

	Group 1 (*n* = 1246)	Group 2 (*n* = 1146)	Group 3 (*n* = 645)	Group 4 (*n* = 1303)	Total (*N* = 4343)
**Marriage**					
Married	284 (22.8%)	523 (45.6%)	487 (75.5%)	1208 (92.8%)	2503 (57.6%)
Non-married	956 (76.7%)	619 (54%)	147 (22.8%)	43 (3.3%)	1767 (40.7%)
Divorce and other	6 (0.5%)	4 (0.4%)	11 (1.8%)	51 (4%)	72 (1.7%)
**Fertility**					
No children	1069 (85.8%)	882 (77%)	288 (44.7%)	142 (10.9%)	2383 (54.9%)
Have children	177 (14.2%)	264 (23%)	357 (55.3%)	1161 (89.1%)	1960 (45.1%)
**Education**					
Specialized school	164 (13.2%)	59 (5.1%)	15 (2.3%)	37 (2.1%)	275 (6.3%)
Specialty	752 (60.4%)	503 (43.9)	134 (20.8)	251 (19.3%)	1641 (37.8%)
B.A.	321 (25.8%)	578 (50.4)	490 (76%)	1008 (77.4%)	2399 (55.2%)
Master	9 (0.7%)	6 (0.5%)	6 (0.9%)	7 (0.5%)	28 (0.6%)
**Major**					
Preschool education	812 (65.2%)	743 (64.8%)	481 (74.6%)	1074 (82.4%)	3111 (71.6%)
Education	154 (12.4%)	162 (14.1%)	130 (20.2%)	182 (14%)	630 (14.5%)
Non-education	280 (22.5%)	241 (21%)	34 (5.3%)	46 (3.5%)	602 (13.9%)
**Title**					
Three class	801 (64.3)	230 (20.1%)	84 (13%)	88 (6.8%)	1204 (27.7%)
Two class	28 (2.2%)	26 (21.1)	59 (9.1%)	545 (41.8%)	659 (15.2%)
The first class	119 (9.6)	358 (31.2%)	338 (52.4%)	573 (44%)	1389 (32%)
High	93 (7.5%)	242 (21.1%)	142 (22%)	90 (6.9%)	567 (13.1%)
Very high	204 (16.4%)	290 (25.3%)	22 (3.4%)	6 (0.5%)	522 (12%)
**Position**					
High	7 (0.6%)	4 (0.3%)	2 (0.3%)	18 (1.4%)	31 (0.7%)
Middle	8 (0.6%)	22 (1.9%)	25 (0.3%)	145 (11.1%)	200 (4.6%)
Low	1231 (98.8%)	1120 (97.7%)	618 (95.8%)	1140 (87.5%)	94.7%

### Procedure

The data were collected in Spring 2016, and this research was a part of a larger cross-sectional study examining mental health and well-being in teachers. All questionnaires consisted of 200 questions and required approximately 40 min to complete. The questionnaires were compiled using an electronic program and released through the Beijing Preschool Education Comprehensive Service Platform, which was operated by the government, and the main users of this platform are preschool teachers and administrators. The electronic questionnaires were distributed to preschools and were answered voluntarily and anonymously by the preschool teachers. And in the introduction of this questionnaire, the participants are informed that all the information about the participants will be kept confidential and only be used for research purposes. To attend the survey, the only personal identity information that the participants are required to offer is their telephone number. The participants were asked to respond to the questionnaires through mobile phones or computers online. After completing the questionnaire, the participants drew prizes from 5 to 100 yuan, with a winning rate of 80%. In addition, before the survey, the participants do not know the reward, and after all the questions were completed, the web program automatically went to a lottery page, and then the participants have the opportunity to draw.

The participants were told that these questionnaires were used to understand their feelings and common problems at work, and they were not aware of the intention of the present study. This study was approved by the Ethics Committee of the School of Preschool Education, Capital Normal University.

### Measures

#### The Chinese Version of the Symptom Checklist 90

The Symptom Checklist 90 (SCL-90) is a 90-item multidimensional questionnaire designed to screen for a broad range of psychological problems in both clinical and non-clinical populations (Derogatis, 1992). Feng and Zhang (2001) developed the Chinese version of the SCL-90 (SCL-90-C). Similar to the SCL-90, the SCL-90-C measures participants’ self-reported psychopathological features on nine subscales (each subscale comprises 6–13 items), including somatization (SOM; e.g., faintness or dizziness), obsessive-compulsiveness behavior (O-C; e.g., unwanted thoughts or ideas that will not leave your head), interpersonal sensitivity (I-S; e.g., feeling critical of others), depression (DEP; e.g., feeling low in energy or slowed down), anxiety (ANX; e.g., nervousness or shakiness inside), hostility (HOS; e.g., feeling easily annoyed or irritated), phobic anxiety (PHOB; e.g., feeling afraid in open spaces or on the street), paranoid ideation (PAR; e.g., feeling that most people cannot be trusted), and psychoticism (PSY; e.g., the idea that someone else can control your thoughts). Each question is rated according to how much the individual was bothered by the item in the last week on a 5-point Likert scale (0 = “not at all,” 1 = “a little bit,” 2 = “moderately,” 3 = “quite a bit,” and 4 = “extremely”). The scores for the nine subscales were classified into three categories: average score < 1, 1 ≤ average score < 2, and average score ≥ 2, which corresponded to “not at all,” “a little bit,” and “moderately, quite a bit or extremely,” respectively.

The SCL-90-C has been used in various contexts and was proved to have adequate reliability and validity among non-clinical populations in China, including within educational settings (Fan et al., 2016; Yang et al., 2019). Despite the relative large amount of items in SCL-90-C, it can detect multiple symptoms of mental health. In the present study, the SCL-90-C demonstrated adequate internal consistency for all dimensions of the questionnaire (greater than 0.74). In addition, a confirmatory factor analysis was conducted using AMOS 4.0 (Arbuckle and Wothke, 1999) to evaluate how well the specified models describe the present data. The results suggested that the SCL-90-C showed adequate structural validity, χ^2^/df = 4.66, normed fit index (NFI) = 0.99, comparative fit index (CFI) = 0.001, Tucker–Lewis index (TLI) = 0.007, and root mean square error of approximation (RMSEA) = 0.036.

#### The Chinese Version of the Interpersonal Reactivity Index

The Chinese version of the interpersonal reactivity index (IRI; Davis, 1983) developed by Huang and Su (2013) was used to assess the empathy of the participants. The IRI-C is a 28-item self-report questionnaire that measures different dimensions of empathy; it comprises four 7-item subscales. The empathic concern subscale was designed to examine one’s capacity to experience feelings of warmth, compassion, and concern toward another person in need (e.g., “I often have tender, concerned feelings for people less fortunate than me”). The personal distress subscale was designed to examine an individual’s own negative emotions as he or she responds to stressful interpersonal situations (e.g., a reversed item, “When I see someone get hurt, I tend to remain calm”). The perspective taking subscale assesses attempts to adopt others’ points of view (e.g., “I try to look at everybody’s side of a disagreement before I make a decision”). The fantasy subscale was designed to examine the likelihood that an individual identifies with a fictional character (e.g., “I really get involved with the feelings of the characters in a novel”). The participants were asked to report on a 5-point Likert scale ranging from 1 (does not describe with me) to 5 (describes with me very well). All of the subscales demonstrated adequate internal reliability, with alpha values of 0.77, 0.82, 0.80, and 0.81 (for empathic concern, personal distress, perspective taking, and fantasy, respectively). Structural validity was examined using AMOS 4.0 (Arbuckle and Wothke, 1999), and the results indicated that the fitness was good, CMIN = 19,481.33, df = 4024, χ^2^/df = 3.69, NFI = 0.97, CFI = 0.001, TLI = 0.008, and RMSEA = 0.043.

## Results

The descriptive analyses of the participants’ mental health and empathy dimensions are shown in Table 2. In addition to the descriptive statistics, three sets of analyses were conducted. First, using *T* tests and multivariate analysis of variance (MANOVA), the characteristics of the participants’ mental health condition were examined. Second, the levels of the participants’ empathy dimensions were examined. Finally, correlation and regression analyses were carried out to investigate the relationship between preschool teachers’ empathy and mental health and in which the role of teaching experience plays.

**TABLE 2:** The descriptive statistics of scores of mental health symptoms and empathy and the ANOVA analysis across the working years groups.

	Group 1 (*n* = 1246)		Group 2 (*n* = 1146)		Group 3 (*n* = 645)		Group 4 (*n* = 1303)		ANOVAs across the working time groups	Total (*N* = 4343)	
	*M*	SD	*M*	SD	*M*	SD	*M*	SD		*M*	SD
**Mental health symptoms**											
SOM	1.61	0.64	1.82	0.73	1.98	0.78	2.15	0.82	*F*(3,4336) = 119.01**	1.88	0.77
O-C	1.87	0.68	2.03	0.72	2.15	0.77	2.25	0.80	*F*(3,4336) = 60.07**	2.07	0.76
I-S	1.64	0.63	1.76	0.66	1.85	0.74	1.97	0.74	*F*(3,4336) = 51.33**	1.80	0.70
DEP	1.58	0.64	1.75	0.69	1.86	0.78	2.00	0.78	*F*(3,4336) = 76.14**	1.79	0.74
ANX	1.56	0.59	1.70	0.67	1.80	0.74	1.91	0.74	*F*(3,4336) = 60.46**	1.74	0.70
HOS	1.52	0.61	1.70	0.68	1.80	0.73	1.95	0.79	*F*(3,4336) = 82.99**	1.74	0.72
PHOB	1.41	0.57	1.51	0.61	1.58	0.65	1.64	0.70	*F*(3,4336) = 30.34**	1.53	0.64
PAR	1.50	0.60	1.62	0.62	1.69	0.67	1.83	0.70	*F*(3,4336) = 55.44**	1.66	0.66
PSY	1.51	0.56	1.61	0.58	1.69	0.63	1.80	0.68	*F*(3,4336) = 52.28**	1.65	0.63
**Empathy**											
FS	3.00	0.58	3.04	0.60	3.05	0.60	2.99	0.58	*F*(3,4336) = 2.81*	3.02	0.59
EC	3.69	0.50	3.66	0.52	3.70	0.54	3.62	0.51	*F*(3,4336) = 5.30**	3.66	0.52
PT	3.61	0.60	3.53	0.59	3.56	0.62	3.50	0.57	*F*(3,4336) = 7.48**	3.55	0.59
PD	2.73	0.54	2.84	0.53	2.85	0.57	2.89	0.55	*F*(3,4336) = 20.10**	2.83	0.55

*p < 0.05, **p < 0.01. SOM, somatization; O-C, obsessive-compulsiveness; I-S, interpersonal sensitivity; DEP, depression; ANX, anxiety; HOS, hostility; PHOB, phobic anxiety; PAR, paranoid ideation; PSY, psychoticism; FS, fantasy; EC, empathic concern; PT, perspective taking; PD, personal distress.

### Preschool Teachers’ Mental Health

According to Table 2, the sample means for each subscale of the SCL-90-C were between 1 and 2, indicating that the preschool teachers exhibited mental health symptoms at levels between “a little bit” and “moderately.”

**FIGURE 1:**
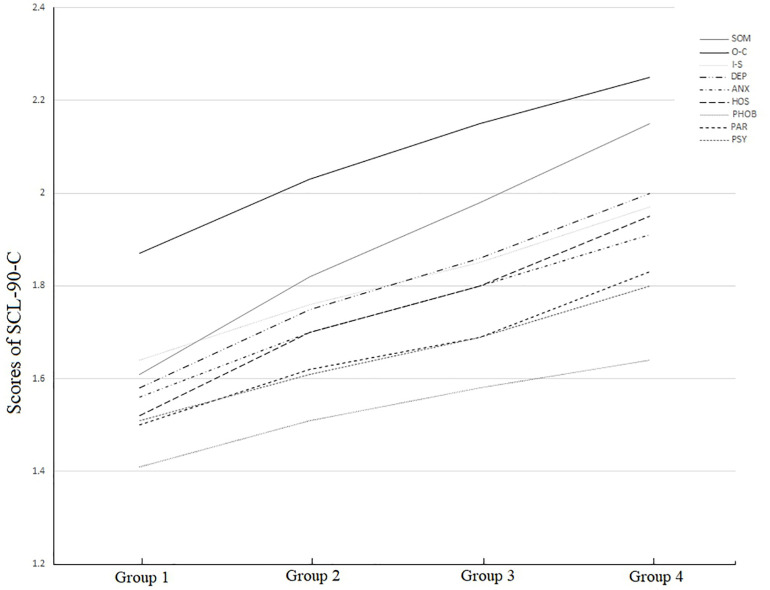
The SCL-90-C scores of the preschool teachers form the four teachering experience groups.

To profile the teachers’ performance on SCL-90-C, a 9 (types of mental health symptoms) × 4 (teaching experience groups) repeated measures MANOVA was performed. Among the variables, the category of mental health symptoms was within-subject variables, and the participants’ teaching experience group was the between-subject variable. The results are shown in Figure 1. The sphericity assumption for repeated measures MANOVA was violated: Mauchly’s *W*_(__35__)_ = 0.341, χ^2^_(__2__)_ = 4620.12, *p* < 0.001. Because violating this assumption inflates the Type I error rate, we used the Huynh–Feldt epsilon to adjust the degrees of freedom and provide a more accurate Type I error rate (Stevens, 2002). The results indicate that there are significant interactive effects between all the nine mental health symptoms and the four teaching experience groups. The interactive effect between the symptoms and teaching experience was significant, *F*(24,4297) = 23.13, *p* < 0.001, $\eta_{\text{p}}^{2}=0.016$, *d* = 1.0. Next, two sets of simple-effect analyses were conducted to analyze the interactive effect from two perspectives.

In the first simple-effect analysis, nine ANOVAs were used to determine the changing characteristics of the nine mental health symptoms across the four teaching experience groups. The results indicated that for all the nine mental health symptoms, there were significant differences between each experience groups, and with the teaching experience increased, all the nine symptoms increased (see Table 2).

In the second simple-effect analysis, we split the data into four teaching experience groups and conducted repeated measures across the nine symptoms in each group. The results suggest that in each of the four teaching experience groups, obsessive-compulsiveness behavior, interpersonal sensitivity, somatization, and depression are the four most serious symptoms. Although in each teaching experience group obsessive-compulsiveness behavior is the most serious symptom, there were some slight differences for the other symptoms: in the group with less than 2 years of teaching experience, somatization was the second most serious symptom, and interpersonal sensitivity was the third most serious problem; in the groups with 2–5 and 5–10 years of teaching experience, the severity of somatization increased and ranked as the second most serious symptom for preschool teachers, and in this teaching experience group, depression was as serious as interpersonal sensitivity and ranked as the third most serious symptom. In the two groups with more than 5 years of teaching experience, the severity of depression outweighed the severity of interpersonal sensitivity and was the third most serious mental health symptom.

In short, the teachers in the present study exhibited mental health symptoms at levels between “a little bit” and “moderately,” and they scored higher than the Chinese norm for all the nine mental health symptoms. The most serious symptoms in preschool teachers are obsessive-compulsiveness behavior, interpersonal sensitivity, somatization, and depression. The severity of all these symptoms increased with experience, and it seems that somatization and depression’s severity grew faster.

### Preschool Teachers’ Empathy

A 4 (dimensions of empathy) × 4 (teaching experience groups) repeated measures MANOVA was performed to profile the teachers’ empathy, and among the variables, the empathy dimensions were within-subject variables, and the participants’ teaching experience group was the between-subject variable. The sphericity assumption for repeated measures MANOVA was violated: Mauchly’s *W*_(__5__)_ = 0.585, χ^2^_(__2__)_ = 2300.11, *p* < 0.001. The results indicate that there are both significant main effects of empathy dimension and interactive effects between dimensions of empathy and teaching experience, for empathy dimension, *F*(3,4297) = 3205.34, *p* < 0.001, $\eta_{\text{p}}^{2}=0.427$, *d* = 1.0; and for interactive effect, *F*(9,4296) = 15.865, *p* < 0.001, $\eta_{\text{p}}^{2}=0.011$, *d* = 1.0.

In the following simple-effect analyses, four ANOVAs (see Table 2) were conducted to compare levels of empathy across the four teaching experience groups, and the results suggested that the four dimensions of empathy showed different changing trends across the four teaching experience periods. For fantasy, no difference was found between any of the teaching experience groups, but for empathic concern, the first (*mean difference* = 0.07, *p* < 0.01) and third teaching experience groups (*mean difference* = 0.08, *p* < 0.01) had significantly higher scores than the fourth group. For perspective taking, the first teaching experience group had higher scores than the second (*mean difference* = 0.08, *p* < 0.01) and fourth teaching experience groups (*mean difference* = 0.11, *p* < 0.01). For personal distress, the first teaching experience group had significantly lower scores than the other three groups (*mean differences* = −0.12, −0.12, and −0.16, *p*s < 0.01). It seems that in the preschool teachers, empathic concern and perspective taking showed a general decreasing trend, whereas personal distress showed an increasing trend.

### The Role of Empathy in Preschool Teachers’ Mental Health

Pearson correlations were conducted to explore the relationship between dimensions of empathy and mental health symptoms (see Table 3). On the basis of correlation analyses, two sets of hierarchical regression analyses were conducted to determine whether empathy predicts mental health symptoms in preschool teachers. The first set of regression analyses used the nine mental health symptoms as dependent variables (see Table 4). For these hierarchical regressions, the demographic variables were entered in Step 1, the length of teaching experience was entered in Step 2, and the four variables of empathy were entered in Step 3. For all nine hierarchical regressions, the models with the set of population measures were significant, *F*s ≥ 19.33, *p*s < 0.001, and *R*^2^s ≥ 0.019, and we found that age significantly predicted all nine mental health symptoms. When the variable for preschool teaching experience was entered in the second step, with the exception of somatization, age did not account for any additional variance in the models, 0.02 ≤ Δ*R*^2^ ≤ 0.09, 0.018, *F* (change) ≥ 16.75, *p* < 0.001. In addition, in this step, teaching experience positively predicted all mental health symptoms, even after the effect of age was controlled. In the third set of models, the four empathy dimensions were entered and showed different predictive patterns for the nine mental health symptoms. On the one hand, both fantasy and personal distress positively predicted the nine mental health symptoms; thus, it seems that the more an individual is prone to fantasy or personal distress, the more susceptible he or she is to these mental health symptoms. On the other hand, empathic concern and perspective taking negatively predicted the nine mental health symptoms; thus, it appears that these two components are protective factors for mental symptoms.

**TABLE 3:** Pearson correlations between participants’ demographic variables, mental health symptoms, and dimensions of empathy.

	2	3	4	5	6	7	8	9	10	11	12	13	14	15	16	17	18	19
**Demographic variables**																		
Age	0.81**	0.60**	0.72**	0.31**	0.19**	0.25**	0.16**	0.14**	0.19**	0.16**	0.16**	0.12**	0.15**	0.16**	−0.11**	−0.03*	−0.03*	0.05**
TE		0.50**	0.59**	0.25**	0.15**	0.25**	0.17**	0.16**	0.19**	0.18**	0.19**	0.13**	0.17**	0.17**	−0.05**	−0.05**	−0.06**	0.08**
Marriage			0.65**	0.34**	0.11**	0.19**	0.12**	0.11**	0.14**	0.12**	0.14**	0.08**	0.12**	0.11**	−0.03*	–0.01	–0.02	0.05**
Fertility				0.30**	0.11**	0.20**	0.14**	0.13**	0.16**	0.14**	0.15**	0.10**	0.13**	0.13**	−0.06**	−0.03*	−0.04**	0.06**
Education					0.16**	0.20**	0.16**	0.13**	0.16**	0.14**	0.17**	0.08**	0.14**	0.14**	–0.00	–0.01	–0.01	0.08**
Income						0.04**	0.03	0.02	0.04**	0.03	0.03*	0.02	0.03*	0.04**	–0.02	–0.02	0.00	–0.01
**Mental health symptoms**																		
SOM							0.84**	0.79**	0.85**	0.87**	0.79**	0.75**	0.76**	0.82**	0.20**	0.07**	−0.09**	0.48**
O-C								0.87**	0.90**	0.88**	0.81**	0.78**	0.81**	0.85**	0.38**	0.11**	−0.09**	0.57**
I-S									0.91**	0.89**	0.83**	0.83**	0.89**	0.90**	0.37**	0.06**	−0.17**	0.57**
DEP										0.92**	0.85**	0.82**	0.87**	0.90**	0.35**	0.05**	−0.16**	0.55**
ANX											0.84**	0.85**	0.85**	0.90**	0.36**	0.05**	−0.14**	0.55**
HOS												0.77**	0.85**	0.84**	0.32**	0.01	−0.22**	0.54**
PHOB													0.79**	0.83**	0.29**	–0.01	−0.17**	0.49**
PAR														0.88**	0.32**	–0.02	−0.21**	0.49**
PSY															0.34**	0.02	−0.17**	0.53**
**Empathy**																		
FS																0.43**	0.20**	0.53**
EC																	0.60**	0.27**
PT																		−0.06**
PD																		

*p < 0.05, **p < 0.01. TE, teaching experience; SOM, somatization; O-C, obsessive-compulsiveness; I-S, interpersonal sensitivity; DEP, depression; ANX, anxiety; HOS, hostility; PHOB, phobic anxiety; PAR, paranoid ideation; PSY, psychoticism; FS, fantasy; EC, empathic concern; PT, perspective taking; PD, personal distress.

**TABLE 4:** Hierarchy regressions predicting the preschool teachers’ mental health symptoms.

		Model 1					Model 2					Model 3							
		Age	Gender	Marriage	Education	Age	Gender	Marriage	Education	WY	Age	Gender	Marriage	Education	WT	FS	EC	PT	PD
SoM	B	0.02	–0.19	0.03	0.16	0.01	–0.14	0.02	0.16	0.01	0.01	–0.03	0.01	0.12	0.01	0.21	–0.08	–0.08	0.54
	β	0.20	–0.05	0.02	0.13	0.10	–0.03	0.02	0.13	0.13	0.16	–0.01	0.01	0.10	0.07	0.16	–0.05	–0.06	0.38
	*t*	11.10**	−3.15**	1.09	8.24**	3.66**	−2.34**	0.93	8.29**	5.22**	6.70**	–0.55	0.54	7.25**	3.12**	9.91**	−3.10**	−3.70**	24.65**
O-C	B	0.01	–0.08	0.02	0.14	0.00	–0.04	0.02	0.14	0.01	0.01	0.10	0.00	0.09	0.00	0.24	–0.08	–0.09	0.65
	β	0.12	–0.02	0.02	0.11	0.03	–0.01	0.01	0.11	0.11	0.10	0.02	0.00	0.08	0.03	0.19	–0.05	–0.07	0.47
	*t*	6.17**	–1.30	0.83	7.06**	1.15	–0.67	0.71	7.09**	4.09**	4.59**	2.03*	0.20	5.85**	1.46	12.25**	−3.04**	−4.31**	32.19**
I-S	B	0.01	–0.06	0.01	0.10	0.00	–0.02	0.01	0.10	0.01	0.01	0.10	–0.01	0.06	0.00	0.24	–0.09	–0.16	0.60
	β	0.11	–0.01	0.01	0.09	0.02	0.00	0.01	0.09	0.12	0.09	0.03	0.00	0.05	0.04	0.20	–0.07	–0.13	0.47
	*t*	5.98**	–0.97	0.47	5.39**	0.63	–0.26	0.34	5.42**	4.63**	4.22**	2.13*	–0.30	3.82**	1.78^†^	12.95**	−3.93**	−8.49**	31.80**
DEP	B	0.01	–0.07	0.02	0.13	0.01	–0.03	0.02	0.13	0.01	0.01	0.08	0.01	0.09	0.00	0.23	–0.10	–0.15	0.62
	β	0.14	–0.02	0.02	0.11	0.05	–0.01	0.01	0.11	0.11	0.12	0.02	0.00	0.07	0.04	0.18	–0.07	–0.12	0.46
	*t*	7.67**	–1.22	0.91	6.90**	1.88^†^	–0.53	0.78	6.94**	4.49**	5.50**	1.68	0.29	5.68**	1.71^†^	11.79**	−4.13**	−7.53**	30.98**
ANX	B	0.01	–0.09	0.01	0.11	0.00	–0.05	0.01	0.11	0.01	0.01	0.06	0.00	0.07	0.00	0.22	–0.13	–0.10	0.59
	β	0.12	–0.02	0.01	0.10	0.02	–0.01	0.01	0.10	0.13	0.10	0.02	0.00	0.06	0.05	0.19	–0.09	–0.09	0.46
	*t*	6.64**	–1.64	0.54	6.03**	0.82	–0.87	0.40	6.06**	4.97**	4.21**	1.37	–0.16	4.56**	2.33*	12.22**	−5.53**	−5.38**	31.08**
HOS	B	0.01	–0.08	0.05	0.14	0.00	–0.02	0.05	0.14	0.01	0.00	0.07	0.04	0.10	0.01	0.20	–0.11	–0.21	0.59
	β	0.10	–0.02	0.04	0.12	–0.04	0.00	0.04	0.12	0.17	0.03	0.02	0.03	0.08	0.09	0.17	–0.08	–0.17	0.45
	*t*	5.15**	–1.33	2.26*	7.53**	–1.38	–0.33	2.07*	7.59**	6.60**	1.52	1.48	1.90	6.52**	4.10**	1.83**	−4.51**	−1.85**	3.43**
PHOB	B	0.01	–0.10	0.00	0.05	0.00	–0.07	0.00	0.05	0.01	0.01	0.01	–0.01	0.01	0.00	0.15	–0.17	–0.10	0.52
	β	0.10	–0.03	0.00	0.05	0.03	–0.02	0.00	0.05	0.09	0.09	0.00	–0.01	0.01	0.02	0.14	–0.14	–0.09	0.44
	*t*	5.43**	−1.85^†^	0.20	2.97**	1.00	–1.29	0.09	2.99**	3.62**	3.83**	0.15	–0.38	0.95	0.78	8.67**	−7.56**	−5.40**	28.25**
PAR	B	0.01	0.03	0.03	0.10	0.00	0.07	0.02	0.10	0.01	0.01	0.15	0.01	0.07	0.01	0.24	–0.15	–0.17	0.47
	β	0.11	0.01	0.02	0.10	–0.01	0.02	0.02	0.10	0.15	0.07	0.04	0.01	0.06	0.06	0.21	–0.11	–0.15	0.39
	*t*	5.68**	0.48	1.27	6.03**	–0.32	1.33	1.10	6.07**	5.65**	2.96**	3.31**	0.65	4.76**	2.93**	13.49**	−6.49**	−9.46**	25.42**
PSY	B	0.01	0.04	0.01	0.09	0.00	0.07	0.01	0.09	0.01	0.01	0.16	0.00	0.06	0.00	0.21	–0.13	–0.12	0.50
	β	0.12	0.01	0.01	0.09	0.03	0.02	0.01	0.09	0.12	0.10	0.05	0.00	0.06	0.04	0.20	–0.11	–0.11	0.44
	*t*	6.53**	0.71	0.57	5.84**	1.05	1.39	0.44	5.87**	4.57**	4.51**	3.84**	–0.12	4.37**	1.74^†^	12.51**	−6.10**	−6.87**	29.35**

*p < 0.05, **p < 0.01. 0.05 † 0.1. SOM, somatization; O-C, obsessive-compulsiveness; I-S, interpersonal sensitivity; DEP, depression; ANX, anxiety; HOS, hostility; PHOB, phobic anxiety; PAR, paranoid ideation; PSY, psychoticism; FS, fantasy; EC, empathic concern; PT, perspective taking; PD, personal distress.

To explore the changing pattern of empathy in mental health symptoms based on teaching experience, hierarchical regressions were conducted in each teaching experience group, with the nine mental health symptoms as the dependent variables (see Table 5). In each hierarchical regression, the demographic variables were entered in the first step, and the four dimensions of empathy were entered in the second step. The results revealed that the predictions of the four dimensions of empathy were different across the four teaching experience groups. As all first models were significant (all *F*s > 29.01, *p*s < 0.001) and to avoid an unwieldy table, we present only the second set of models in Table 5. The results revealed that fantasy and personal distress positively predicted all mental health symptoms in each teaching experience group; however, the roles of empathic concern and perspective taking were different across the four teaching experience groups. Although empathic concern negatively predicted anxiety, phobic, anxiety, paranoid ideation, and psychoticism in all the four teaching experience groups, it selectively negatively predicted somatization, obsessive-compulsiveness behavior, interpersonal sensitivity, depression, and hostility only in participants with more than 5 years teaching experience (in the third or four teaching experience group). In contrast, although perspective taking also negatively predicted the similar symptoms with empathic concern in all teaching experience groups, it only negatively predicted somatization, obsessive-compulsiveness behavior, and phobic anxiety for the participants with teaching experience less than 5 years (in the first and second teaching experience groups). That is to say, empathic concern plays a protective role against mental problems mainly in new preschool teachers, whereas empathic concern plays a protective role mainly in experienced preschool teachers.

**TABLE 5:** Hierarchy regressions predicting the preschool teachers’ mental health symptoms in the four working year groups.

		Group 1			Group 2			Group 3			Group 4		
Model		B	β	*t*	B	β	*t*	B	β	*t*	B	β	*t*
SoM	FS	0.20	0.18	6.08**	0.21	0.18	5.52**	0.20	0.15	3.34**	0.21	0.15	4.68**
	EC	–0.01	–0.01	–0.20	–0.07	–0.05	–1.43	–0.10	–0.07	–1.35	–0.14	–0.09	−2.67**
	PT	–0.15	–0.14	−4.43**	–0.11	–0.09	−2.63*	–0.01	–0.01	–0.14	–0.04	–0.02	–0.79
	PD	0.44	0.37	12.58**	0.51	0.37	11.77**	0.52	0.38	9.02**	0.64	0.44	14.72**
O-C	FS	0.22	0.19	6.65**	0.23	0.19	6.40**	0.27	0.21	5.00**	0.24	0.18	6.02**
	EC	–0.03	–0.02	–0.77	–0.02	–0.01	–0.35	–0.04	–0.03	–0.67	–0.18	–0.11	−3.60**
	PT	–0.14	–0.13	−4.20**	–0.12	–0.10	−3.22**	–0.03	–0.02	–0.50	–0.04	–0.03	–1.07
	PD	0.59	0.47	17.05**	0.63	0.46	15.93**	0.59	0.43	11.23**	0.75	0.52	18.69**
I-S	FS	0.20	0.18	6.41**	0.21	0.19	6.46**	0.31	0.25	6.17**	0.25	0.19	6.65**
	EC	–0.08	–0.07	−2.09*	–0.02	–0.01	–0.43	–0.07	–0.05	–1.10	–0.17	–0.12	−3.70**
	PT	–0.15	–0.14	−4.84**	–0.21	–0.19	−5.87**	–0.17	–0.14	−3.38**	–0.12	–0.10	−3.31**
	PD	0.57	0.49	17.93**	0.55	0.44	15.14**	0.55	0.43	11.28**	0.67	0.50	18.03**
DEP	FS	0.19	0.17	6.09**	0.20	0.17	5.73**	0.29	0.22	5.30**	0.25	0.18	6.17**
	EC	–0.07	–0.05	–1.63	–0.04	–0.03	–0.95	–0.09	–0.06	–1.39	–0.18	–0.12	−3.75**
	PT	–0.16	–0.15	−5.05**	–0.19	–0.16	−5.08**	–0.13	–0.11	−2.55**	–0.11	–0.08	−2.84**
	PD	0.55	0.47	16.75**	0.59	0.45	15.29**	0.58	0.42	10.98**	0.70	0.49	17.70**
ANX	FS	0.20	0.19	6.78**	0.21	0.18	6.09**	0.27	0.22	5.18**	0.24	0.18	6.22**
	EC	–0.07	–0.06	−1.80^†^	–0.10	–0.08	−2.27*	–0.13	–0.10	−2.06*	–0.20	–0.14	−4.44**
	PT	–0.13	–0.13	−4.34**	–0.12	–0.10	−3.18**	–0.09	–0.08	−1.81^†^	–0.08	–0.06	−2.07*
	PD	0.51	0.47	16.92**	0.59	0.46	15.53**	0.56	0.43	11.07**	0.67	0.49	17.67**
HOS	FS	0.17	0.16	5.47**	0.18	0.16	5.29**	0.24	0.20	4.80**	0.23	0.17	5.85**
	EC	–0.06	–0.05	–1.48	–0.05	–0.04	–1.14	–0.14	–0.10	−2.29*	–0.17	–0.11	−3.58**
	PT	–0.21	–0.20	−6.61**	–0.28	–0.24	−7.58**	–0.17	–0.14	−3.37**	–0.19	–0.14	−4.87**
	PD	0.48	0.43	15.02**	0.59	0.46	15.76**	0.57	0.45	11.63**	0.69	0.49	17.52**
PHOB	FS	0.14	0.15	4.93**	0.13	0.13	3.97**	0.18	0.16	3.69**	0.18	0.14	4.71**
	EC	–0.08	–0.07	−2.16*	–0.10	–0.08	−2.28*	–0.20	–0.17	−3.43**	–0.30	–0.22	−6.74**
	PT	–0.13	–0.13	−4.24**	–0.14	–0.14	−4.05**	–0.06	–0.06	–1.29	–0.06	–0.05	–1.64
	PD	0.47	0.45	15.30**	0.50	0.43	13.82**	0.48	0.42	10.43**	0.59	0.47	16.15**
PAR	FS	0.19	0.19	6.36**	0.22	0.21	6.84**	0.28	0.25	5.80**	0.29	0.23	7.64**
	EC	–0.10	–0.08	−2.54**	–0.08	–0.07	−1.94*	–0.15	–0.12	−2.56**	–0.24	–0.17	−5.32**
	PT	–0.18	–0.18	−5.88**	–0.22	–0.21	−6.30**	–0.17	–0.15	−3.56**	–0.13	–0.11	−3.61**
	PD	0.45	0.41	14.01**	0.45	0.38	12.71**	0.43	0.37	9.23**	0.52	0.40	13.94**
PSY	FS	0.20	0.20	7.12**	0.18	0.18	5.95**	0.23	0.22	5.13**	0.23	0.19	6.53**
	EC	–0.08	–0.07	−2.21*	–0.08	–0.07	−2.13*	–0.10	–0.08	−1.82^†^	–0.22	–0.17	−5.28**
	PT	–0.13	–0.14	−4.46**	–0.14	–0.14	−4.34**	–0.13	–0.12	−2.89**	–0.09	–0.08	−2.65**
	PD	0.47	0.45	16.24**	0.48	0.43	14.38**	0.45	0.40	10.27**	0.58	0.47	16.79**

*p < 0.05, **p < 0.01. 0.05 † 0.1. SOM, somatization; O-C, obsessive-compulsiveness; I-S, interpersonal sensitivity; DEP, depression; ANX, anxiety; HOS, hostility; PHOB, phobic anxiety; PAR, paranoid ideation; PSY, psychoticism; FS, fantasy; EC, empathic concern; PT, perspective taking; PD, personal distress.

## Discussion

The present study examined the characters of mental health and empathy in Chinese preschool teachers. Moreover, this study revealed that empathy played both protective and risk roles in preschool teachers’ mental health, and these roles were affected by preschool teachers’ teaching experience.

### Preschool Teachers’ Mental Health

By examining mental health in a sample covering a wide range of teaching experiences, the present study reveals the general condition and dynamic aspect of Chinese preschool teachers’ mental health.

First, the results indicate that as teachers’ experience increases, preschool teachers exhibit a trend toward increases in mental health symptoms. This result was supported by a meta-analysis, which indicated that during the period of 1998–2013, Chinese teachers’ mental health becomes worse (Yang et al., 2019). However, despite its extreme importance, the mental status of teachers is still worrying.

In addition, the characteristics of the nine symptoms suggested that for each of the four teaching experience groups, obsessive-compulsiveness behavior, somatization, depression, and interpersonal sensitivity were the most serious symptoms, with the severity of somatization and depression increasing faster. Among these symptoms, obsessive-compulsiveness behavior is the most prominent mental health problems for preschool teachers with various teaching experience. It seems that obsessive-compulsiveness behavior is a typical mental problem in teaching professionals, as obsessive-compulsiveness behavior was also found as the most serious mental health problem among Chinese primary and high school teachers (Zeng and Li, 2011). Interpersonal sensitivity is also a typical mental problem especially for the new preschool teachers. Teaching, by nature, is a complex interpersonal interaction, and new teachers have to rebuild their interpersonal relationships, which may contribute to the preschool teachers’ interpersonal sensitivity. In addition, the increase in severity of somatization and depression with teaching experience may reflect the process of emotional exhaustion and job burnout, as these two indexes are sensitive to stress and emotional labor (Seery and Corrigall, 2009), which are both typical characters in preschool education (Miller et al., 2019).

### Preschool Teachers’ Empathy

The present study found that among preschool teachers, the four dimensions of empathy showed different patterns as teaching experience increased: fantasy remained stable, empathic concern and perspective taking showed a slow decreasing trend, and personal distress showed an increasing trend.

According to a research on student preschool teacher (Huang et al., 2018), the mechanisms underlying the changing pattern of empathy are dehumanization and detachment, which are indexes of job burnout (Haslam and Loughnan, 2014). Preschool teaching typically involves emotional labor, of which emotional engagement is a core part (Philipp and Schüpbach, 2010). Exposure to children’s emotions for an extended period could decrease preschool teachers’ sensitivity to emotional cues, and empathy, if it is too strong or lasts too long, could increase the risk of emotional exhaustion (Wróbel, 2013; Wacker and Dziobek, 2018). Accordingly, the changes observed in the preschool teachers may reflect a protective mechanism.

### The Relationship Between Empathy and Mental Health

The present study found a relatively complex relationship between empathy and mental health in preschool teachers. These results only partially supported our first hypothesis. This study indicated that empathy dimensions correlate with mental health differently; however, this study also suggested that fantasy and personal distress are risk factors to preschool teachers’ mental health, whereas empathic concern and perspective taking play protective roles in preschool teachers’ mental health. In addition, the second hypothesis was supported as the result indicated that the relationships between dimensions of empathy and mental health symptoms were different across the four teaching experience groups.

#### Fantasy and Personal Distress Are Risk Factors to Preschool Teachers’ Mental Health

First, this study revealed that fantasy and personal distress predicted more mental health symptoms in preschool teachers with various teaching experience. In accordance with some previous studies, the present research also proves that, as an important interpersonal skill set, empathic tendencies can also be “risk strengths” (Bubandt and Willerslev, 2015) and may confer risk for internalizing problems (Tone and Tully, 2014).

Among these findings, the result suggesting that personal distress is a risk factor to mental health was observed by many previous studies. For example, personal distress was found to significantly predict the perceived stress in medical staff (Ortiz Barón et al., 2018) and teachers (Vucinic et al., 2020).

However, our result also suggested that fantasy increased susceptibility to mental health symptoms, and this result was somewhat surprising, because fantasy is usually regarded as a cognitive component of empathy (Wu et al., 2012; Karyagina et al., 2017), and some previous research has found the positive function against mental health-related problems. For example, Karyagina et al. (2017) have found that in nurses, both perspective taking and fantasy negatively correlated with professional burnout, which is also a typical index of mental health. However, in the present study, fantasy is proved to be a risk factor to mental health symptoms. An explanation is provided by the Karyagina et al. (2017) study, which investigated the medical staff, who are often exposed to stronger emotional expressions, whereas the participants in this study were teachers whose working environment was different from that of the medical staff. Another recent research conducted in college sample also found that both fantasy and personal distress are negatively associated with neuroticism dimension in Eysenck Personality Questionnaire and positively correlated with the individual’s trends of feeling anxiety and stress (Neumann et al., 2016).

This result concerning the risk role of fantasy reminds us to reconsider and interpret carefully the nature and function of fantasy. Fantasy is usually defined as an automatic tendency to imaginatively transpose oneself into fictional situations (Keaton, 2017). According to the description of the items in the IRI (Davis, 1983), fantasy is a trend of automatically imitating or mimicking a fictional character. Therefore, individuals high in fantasy are more likely to experience similar negative affect when facing others’ emotional suffering. However, more especially designed research is needed to find out the nature and functions of fantasy.

#### Empathic Concern and Perspective Taking Are Protective Factors in Preschool Teachers’ Mental Health

This study also indicated that empathic concern and perspective taking were negatively related with preschool teachers’ mental health problems. These results imply that these two empathy components may be protective factors for preschool teachers’ mental health. The positive effects of empathic concern and perspective taking were supported by a large number of research from both medical (Thirioux et al., 2016) and educational fields (Ahmetoglu and Acar, 2016; Bullough, 2019). This result was also supported by a meta-analysis (Longmire and Harrison, 2016) of 269 independent samples, which found positive effects of perspective taking and empathic concern on various outcome domains at work. According to the classical theoretical models of Preston and de Waal (2002) and Decety and Lamm (2006), the mechanism underlying the positive effects of empathic concern and perspective taking may be that both of these two empathy components are based on the self-other distinction and are regulated by the up–down processes, and therefore, individuals high in these two empathy components are less likely to be overwhelmed by the others’ distress, more open to the person in distress, and consequently more resistant to mental health problems (Thirioux et al., 2016; Wacker and Dziobek, 2018).

Moreover, this study also found a difference between the roles of empathic concern and perspective taking in preschool teachers’ mental health during their working years. This result suggested the protective effects of perspective taking were greater than those of empathic concern. In this study, perspective taking negatively predicted all mental health problems across almost all the four working year groups, whereas empathic concern only negatively predicted symptoms such as phobic anxiety, paranoid ideation, and psychoticism. In addition, empathic concern has a protective effect only in experienced preschool teachers with longer teaching experience. The stronger protective effects of perspective taking were also suggested by other research. For example, Lamothe et al. (2014) found that perspective taking alone might be a protective factor for burnout, whereas empathic concern happens only under the balance of perspective taking. This may because cognitive empathy requires effort to understand the person in distressing experiences while keeping a certain affective distance, whereas empathic concern, by nature, is an emotional component that involves a non-conscious sharing when facing others’ suffering.

#### How Different Empathy Components Correlated With Mental Health

Overall, our study indicates that as a multidimensional phenomenon, empathy builds upon the cooperation of automatic, emotional, and cognitive processes to cope with the demand raised by environments. For preschool teachers who are surrounded with various emotions and whose job is typically an emotional labor (Philipp and Schüpbach, 2010), different empathy components function differently to help teachers to adapt to their job and keep the balance between self and other (Chiu and Yeh, 2017).

Among the components of empathy, personal distress and fantasy facilitate the overwhelming merging from self to others to feel the others’ feeling, which increases one’s susceptibility to negative emotions and stresses and consequently results in higher risk of stress and mental health problems (Keaton, 2017; Huang et al., 2018). Empathic concern and perspective taking are based on the distinction between self and others, protect one from being overwhelmed by others’ emotion, and consequently help in resisting stress and mental health problems. From this perspective, this study deepened our understanding of the function of empathy and the emotional nature of preschool teaching.

### The Role of Preschool Teaching Experience in the Relationship Between Empathy and Mental Health

The present research also indicates a dynamic relationship between empathic concern, perspective taking, and preschool teachers mental health status across different teaching experience groups. A possible explanation to this result is the psychological nature of teaching experience.

According to Katz’s (1972) theoretical framework, preschool teachers’ development contains four stages: survival, consolidation, renewal, and maturity stage. The most prominent feature of the earlier career stage, such as the survival and consolidation stages, is that teachers cannot break through the self-centeredness and mainly focus on their own survival and adaptation. Preschool teachers have to use the cognitive tool, for example, perspective taking, to understand the feeling and thoughts of others and consequently to adopt the working task and personal relationship. With the increase of teaching experience, preschool teachers enter the stage of renewal and maturity stages; and empathic concern, which is automatic and affective in nature, may help the teachers feel and understand others’ emotions better. From this perspective, this research highlights the role of teaching experience in an emotional context.

## Theoretical and Practical Implications

From the theoretical perspective, this study deepens and broadens our understanding of empathy, mental health, teaching experience, and their relationship. To begin with, this study implies the complicated role of empathy in individuals’ occupations and shed light on the interpersonal nature of empathy (Coutinho et al., 2014). In addition, this study also adds empirical evidence of teachers’ developmental stages (Katz, 1972) by using the context of preschool teaching.

The present study also provides practical implications for teachers’ mental health. First, to minimize the risk of poor mental health in preschool teachers, screening and professional intervention services should be provided at different career stages, especially in preschool teachers with more teaching experience, who may be more susceptible to various mental health symptoms. Second, it is important to take empathy into consideration when designing and implementing intervention programs for teachers’ mental health, and both the positive and negative effects of empathy should be taken into consideration (Erera, 1997; Goroshit and Hen, 2014). According to the present study, empathic concern and perspective taking are protective factors to preschool teachers’ mental health, and intervention programs can facilitate teachers’ mental health by cultivating their empathic concern and perspective taking. Moreover, personal distress and fantasy were found to be potential risk factors underlying preschool teachers’ mental health, so it is also necessary to decrease these two components of empathy.

## Values, Limitations, and Future Directions

This study was an important attempt to integrate the potential factors of teachers’ mental health. The results provide valuable insight into the risk and protective functions of empathy in teachers’ mental health. Furthermore, the Chinese sample of the present study broadens our understanding of this important problem by adding data on non-Western cultures. In addition, the adequate sample size was also an advantage of this research.

This study has several limitations that must be mentioned. First, the cross-sectional design is unable to provide a causal explanation with regard to how empathy affects preservice teachers’ mental health. A longitudinal design or intervention research is needed in the future. Second, the sample in the present study included only public preschool teachers and thus does not represent all preschool teachers. Future research should include not only teachers in public preschools but also teachers in private preschools to ensure a fully representative sample. Finally, the participants’ mental health and empathy scores were based on self-reports. Although the most widely used measures were utilized in this research, societal stereotypes could bias the evaluation of self-reports. Moreover, although the processes in this study were carried out semi-anonymously, the self-reporting nature of the current study means its results may be affected by the socially desirable effect. Therefore, further studies should include multiple methods, including behavior or physiological indicators, to explore the underlying mechanism of preschool teachers’ mental health.

## Data Availability Statement

The datasets generated for this study are available on request to the corresponding author.

## Ethics Statement

The studies involving human participants were reviewed and approved by the Ethic Committees in Capital Normal University. The patients/participants provided their written informed consent to participate in this study.

## Author Contributions

HH and YL performed material preparation and, data collection and analysis. HH wrote the first draft of the manuscript. YS and HH performed the funding acquisition. YS conducted the supervision. All authors contributed to the study conception and design, commented on previous versions of the manuscript and, read and approved the final manuscript.

## Conflict of Interest

The authors declare that the research was conducted in the absence of any commercial or financial relationships that could be construed as a potential conflict of interest.
